# eSIP-Saúde: Mozambique’s novel approach for a sustainable human resources for health information system

**DOI:** 10.1186/s12960-016-0159-y

**Published:** 2016-11-05

**Authors:** Keith P. Waters, Moises Ernesto Mazivila, Martinho Dgedge, Edgar Necochea, Devan Manharlal, Alexandra Zuber, Beatriz de Faria Leão, Debora Bossemeyer, Alfredo E. Vergara

**Affiliations:** 1 Division of Global HIV & TB, Center for Global Health, U.S. Centers for Disease Control and Prevention, 1600 Clifton Road, MS-E77, Atlanta, GA 30329 United States of America; 2 Ministry of Health, Mozambique, Av. Eduardo Mondlane, No. 1008, Maputo, Mozambique; 3 Jhpiego/Johns Hopkins University, 1615 Thames Street, Baltimore, MD 21231 United States of America; 4 Jhpiego Mozambique, Rua Jose Mateus 27, Maputo, Mozambique; 5 CDC Mozambique, JAT Complex 4, 7th Floor, Ave. Zedequias Manganhela 267, Maputo, Mozambique

**Keywords:** Human resources information system (HRIS), Health workforce registry, HIV/AIDS, Data use, Deployment, Staffing, Retirement

## Abstract

**Introduction:**

Over the past decade, governments and international partners have responded to calls for health workforce data with ambitious investments in human resources information systems (HRIS). However, documentation of country experiences in the use of HRIS to improve strategic planning and management has been lacking. The purpose of this case presentation is to document for the first time Mozambique’s novel approach to HRIS, sharing key success factors and contributing to the scant global knowledge base on HRIS.

**Case presentation:**

Core components of the system are a Government of Mozambique (GOM) registry covering all workers in the GOM payroll and a “health extension” which adds health-sector-specific data to the GOM registry. Separate databases for pre-service and in-service training are integrated through a business intelligence tool. The first aim of the HRIS was to identify the following: who and where are Mozambique’s health workers? As of July 2015, 95 % of countrywide health workforce deployment information was populated in the HRIS, allowing the identification of health professionals’ physical working location and their pay point.

HRIS data are also used to quantify chronic issues affecting the Ministry of Health (MOH) health workforce. Examples include the following:

- HRIS information was used to examine the deployment of nurses trained in antiretroviral therapy (ART) vis-à-vis the health facilities where ART is being provided. Such results help the MOH align specialized skill sets with service provision.
- Twenty-five percent of the MOH health workforce had passed the 2-year probation period but had not been updated in the MOH information systems. For future monitoring of employee status, the MOH established a system of alerts in semi-monthly reports.
- As of August 2014, 1046 health workers were receiving their full salary but no longer working at the facilities. The MOH is now analyzing this situation to improve the retirement process and coordination with Social Security.

**Conclusion:**

The Mozambican system is an important example of an HRIS built on a local platform with local staff. Notable models of strategic data use demonstrate that the system is empowering the MOH to improve health services delivery, health workforce allocation, and management. Combined with committed country leadership and ownership of the program, this suggests strong chances of sustainability and real impact on public health equity and quality.

## Background

In the “decade of action on human resources for health (HRH)” launched by the World Health Organization’s 2006 global report [1], significant progress has been made in the development of global health workforce data. Starting with the first sentinel calls by the Joint Learning Initiative [2], a panoply of global calls for workforce data have been made [3–8], and governments and global partners have responded with ambitious investments in national health workforce observatories (led by the World Health Organization (WHO)) and national human resources information systems (HRIS). The US President’s Emergency Plan for AIDS Relief (PEPFAR) alone has invested in the development of over 17 national HRIS [9], mostly in Africa, to improve the strategic planning and management of the health workforce involved in human immunodeficiency virus (HIV) service delivery.

Despite this broad investment, a 2012 systematic review of HRIS development globally found that implementation science and other documentation of country experiences in the strategic use of HR data was lacking and that further development in this area is needed [10]. Since then, only a few papers have been published on HRIS in Sub-Saharan Africa: these cover the impact of Kenya’s HRIS [11–14], the benefits of Uganda’s HRIS [15, 16], and the rollout of an HRIS in Tanzania [17]. A more recent systematic review of HRIS in health care also determined that there is still little published research on the use and impact of HRIS [18].

This case study presents the Mozambique HRIS experience described by the Ministry of Health (MOH) and Jhpiego, the implementing partner developing the HRIS. We make reference to official MOH publications, supported by data from the component HRIS databases analyzed by Jhpiego. The paper is organized as follows: brief background on the health situation in Mozambique and case presentation covering key aspects of the information value cycle: organizational context and information needs, system development and architecture, implementation and rollout, integration with government payroll and training databases, data quality, and data analysis—providing several strong examples of strategic use of data outputs from the HRIS, followed by a discussion section. The purpose is to document the Mozambican HRIS for the first time, sharing key success factors and lessons learned by Mozambique and contributing to the scant global knowledge base on HRIS.

In recent years, Mozambique has seen encouraging improvements in its health indicators, including reductions in maternal, neonatal, and infant mortality, increases in the coverage of immunization and institutional deliveries, reduction of the malaria mortality rate, expansion of access to tuberculosis (TB) treatment, and a significant increase in the number of persons benefiting from antiretroviral treatment (ART) [19]. Also promising is the increase in health workforce density to population during this period: the ratio of doctors, nurses, and midwives increased from 47.9 to 67.3 per 100 000 population from 2005 to 2013 [20]. Key to achieving these improvements has been the sustained action of the MOH, a national environment of economic growth and stability, and, above all, the silent, dedicated, and lasting work of thousands of health workers throughout the country.

The world is aspiring to universal health coverage (UHC) [21], yet it is estimated that 10.3 million additional health workers worldwide are required to close current gaps and ensure UHC delivery [22], and Mozambique ranked 10th from the bottom on the WHO 2010 list of countries with the greatest health workforce deficits [23]. There are just under 40 000^1^ health workers in Mozambique’s public sector (paid from the state budget and “Pro Saude” donor funds), and the sheer deficits across the country are coupled with poor distribution and uneven competency of these health workers, which jeopardize access and quality of health care delivery [19, 24]. Addressing these constraints is more challenging due to the historical lack of available and updated data on the health workforce that decision makers in the MOH could use to make more efficient training, allocation, and management decisions. To address this issue, the MOH, with PEPFAR support, initiated the development of a national HRIS to help improve planning and management of health workers countrywide.

## Case presentation

### Organizational context and information need

The conceptualization and design of the HRIS was based on an assessment of existing systems conducted by the MOH in 2010 [25]. The MOH was aware of the advantages information systems bring to the implementation and monitoring of policies and had identified the need for an integrated HRH information system in its National HRH Development Plan 2008–2015. The MOH considered this assessment a required first step. The assessment found that there were several computerized HRIS operating in the MOH. One of the systems was part of the routine MOH Health Management Information System (HMIS). It was Excel-based and providing periodic information on HRH, by broad categories, available in the health facilities. The other two were part of larger Government of Mozambique (GOM) HR Information Systems: an access-based Personnel Information System (SIP in Portuguese) administrated by the Ministry of State Administration and Public Function (MAEFP), supposed to capture detailed information on personnel, including demographics and staff life-cycle elements (employment status, retirements), and an online Oracle Database of State Employees (eletrônico Cadastro de Agentes e Funcionários do Estado (eCAF)) administrated by the Ministry of Economy and Finance (MEF), related to payroll and linked to other financial and budgetary government systems but with limited information needed for HR management purposes. There were important data inconsistencies among these systems. Moreover, none of them contained up-to-date or complete information, making them ineffective for informing HR decision making.

### HRIS development and system architecture

The MOH and partners US Centers for Disease Control and Prevention (CDC) and Jhpiego decided that, for sustainability reasons, the new MOH HRIS had to be built upon what already existed, be part of the MOH HMIS architecture, and be linked to the larger GOM systems. At the time, the MEF was striving for better control of critical government expenditures and interested in ensuring the coverage and data quality of eCAF. Thus, the MEF provided development capacity, internet communication capability, and technical support to the MOH and made eCAF available, and in return, national-level MOH HR staff became involved in ongoing data verification and system improvement.

**Fig. 1:**
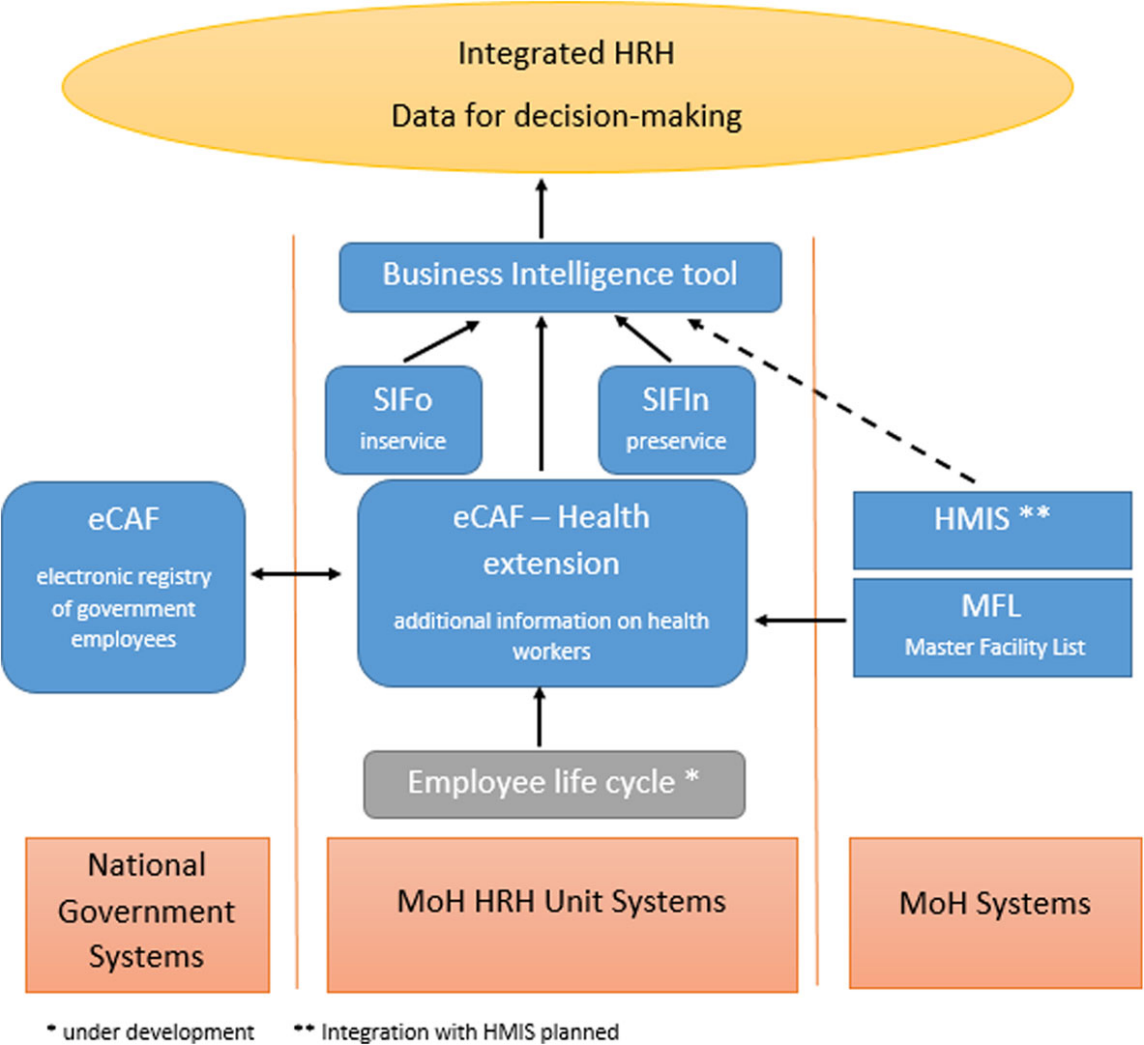
HRIS (eSIP-Saúde) main components and relationships with other MOH and GOM systems

The “concept name” for the Mozambican HRIS became “eSIP-Saúde” (electronic Personnel Information System for health). Figure 1 depicts the existing and envisioned components of the HRIS and its relationships with other MOH and GOM systems. Core components of eSIP-Saúde are eCAF, serving as a national registry covering all workers paid by the GOM, and the eCAF “health extension,” which adds additional health-sector-specific data elements to the registry, including the physical location of workers to the health facility level and professional occupation categories. Both eCAF and the health extension were developed by the MEF’s information technology (IT) unit: Center for Development of Financial Information Systems (CEDSIF). The software was developed by CEDSIF’s technical staff using open source tools and written in Java. The source code is owned by the MEF. CEDSIF comprises approximately 200 staff (working on multiple projects, not only eCAF), conducts requirement-gathering processes, and has system architects, testing, development, and operations departments, plus a well-supported helpdesk.

### Implementation and rollout

In 2011, building upon the online eCAF employee database began—“extending” it to include additional information on personnel required for HRH management. The idea of this initial phase of the HRIS was to answer the following question: who and where are the health workers in the country?

Starting in 2014, work commenced to extend eCAF to also cover health sector workers not paid by government (e.g., paid by non-governmental organizations (NGOs), donors, or foreign governments).

Through CEDSIF, the MEF provides the informatics infrastructure for the HRIS. Data are entered by MOH HR staff directly into eCAF via web browsers in provincial and district offices using the MEF’s private network. As the CDC-funded development partner, Jhpiego staff serve as technical advisors on HRIS to the MOH. As such, Jhpiego, MOH, CEDSIF, and the MAEFP (in charge of the state Personnel Information System) form a technical team collaborating on system integration, electronic data exchange, indicator development, and specifications. MOH and Jhpiego do not have direct access to the databases stored on CEDSIF servers; CEDSIF sends a copy to them each month. The fact that CEDSIF, rather than a donor organization, provides the software development and an important part of the IT infrastructure helps to reduce the costs of the HRIS. Using existing government infrastructure has saved an estimated $367 000 in initial costs: on 184 computers (covering each of the 142 districts, 11 provincial health departments (2 each), and 20 hospitals), at an average of nearly $2000 each including delivery costs, and the construction of a $20 000 data center at the MOH central level. Also saved are additional ongoing costs of maintaining servers, internet connection, and network assistance, each just over $5000 per month, estimated at $187 000 per year.

The rollout of the HRIS nationwide involved several partners at different levels. US government (USG) cooperating agencies working with PEPFAR and other USG funds at the provincial level provided additional support for supervision, training, and quality control. At the central MOH level, the Belgian Technical Cooperation (BTC) provided complementary funding for training and supervision. Regular periodic partners’ meetings serve to plan, coordinate, and align efforts. This cooperation with various other partners has enabled sharing the cost of trainings and led to many more people being trained.

The current phase of HRIS development, in progress from late 2014, covers the health worker lifecycle (such HR functions as promotions, transfers, retirement) to enable more efficient human resources management.

As of July 2015, 95 % of countrywide health workforce deployment information (36 312 out of 38 383 health workers) was populated in the eCAF-health extension, and an existing MOH master facility list was updated to allow the facility-level allocation of workers.

### Integration with payroll

The HRIS allows the identification of the physical location where health professionals are working, as well as their pay point. The HRIS, because it is constructed upon the eCAF database, is integrated with the national payroll system for all government workers (eFOLHA) from which direct bank payments are made.

Approximately 10 % of health workers are paid by non-government sources. Most of these non-government workers (known as “fora de quadro”) are paid from a pooled donor funds basket (“Pro Saude”). Some are paid by other US partners. The private sector in Mozambique is relatively small and not currently included in the HRIS. Initial focus was on health workers paid by the government. The HRIS was then expanded to capture the health workers not paid by the government and by July 2015 contained around 35 % of them as well. Further, in 2015, the HRIS began to capture community health assistants (agentes polivalentes elementares de saúde (APEs))—a cadre of community health workers currently supported by donors and not yet in the government payroll.

### Training databases

Independent databases for pre-service training (Sistema de Informação da Formação Inicial (SIFIn) in Portuguese) and in-service training (Sistema de Informação da Formação Continua (SIFo) in Portuguese) are also part of the HRIS. SIFIn contains comprehensive data on current enrolled and graduating health career students from 13 out of 16 MOH pre-service institutions in all provinces in Mozambique. This includes all classes taken and grades attained for students, and profiles of all tutors allowing analysis of the future pipeline of health workers, teaching quality, and staffing projections. SIFo captures data on in-service training provided to health workers already working in the public health system. Integrated and aggregated reporting of data from all components of the HRIS is done using a business intelligence tool (Pentaho), allowing comprehensive analysis of current staffing trends and deficits, training allocation, and the future supply and demand for health workers.

### Data quality

An online HRIS community of practice (CoP) was established for assessing data quality. All MOH human resources managers at the central and provincial levels, as well as some district-level HR managers and cooperation partners, are part of this CoP. Data quality is constantly improved through the HRIS CoP and the use and verification of data (e.g., data inconsistencies, creation of new health facilities, problems with codification of facilities). The CoP periodically disseminates the achievement of indicators among its members and recognizes best performers—rewards, such as laptops, have sometimes been given to provincial HRH units for achieving coverage and quality targets. Additional measures to improve data quality are being designed and planned, such as “proof of life,”^2^ which is to be implemented by the Ministry of Public Function and CEDSIF, and the verification of data at the district level using a sampling methodology.

Data completeness of the health worker database (the eCAF extension) is assessed against the main eCAF/payroll database monthly. The level of completeness is consistently around 95 %, although this fluctuates slightly (completeness for June 2015 was 96.8 %).

### Data use

Now that the HRIS is populated and the quality of the data closely monitored, more attention is being turned to the information that can be reported from the system and analyses that can inform decision and policy making. The HRIS CoP promotes the use of data at the local level. Provinces such as Cabo Delgado, Tete, Zambézia, Inhambane, Maputo Province, Gaza, and Sofala already use data to produce HRH provincial reports. In 2011, the MOH created the national Health Workforce Observatory, with technical support from the WHO HRH Unit in Geneva. Members of the observatory include different MOH units (the HRH directorate, National Institute of Health, and others), National Institute for Statistics, other ministries, national university/school of medicine, and cooperation partners. The Observatory’s objective is to establish a national platform for health workforce data analysis to improve policy and planning, and it is starting to use HRIS information: semi-monthly HR reports are being produced and published on the MOH website [26], including number of health workers by gender, location (province, district or health facility), cadre, area (urban/rural), health facility type (primary, secondary, tertiary), age, and occupational level, and the ratio of health workers to 100 000 population. The HRIS was used as the main source of information for the 2014 Annual National Statistical Overview of Human Resources for Health in Mozambique. It is also being used to examine in more detail aspects related to deployment and management of HRH.

#### Actual deployment of key providers

**Fig. 2:**
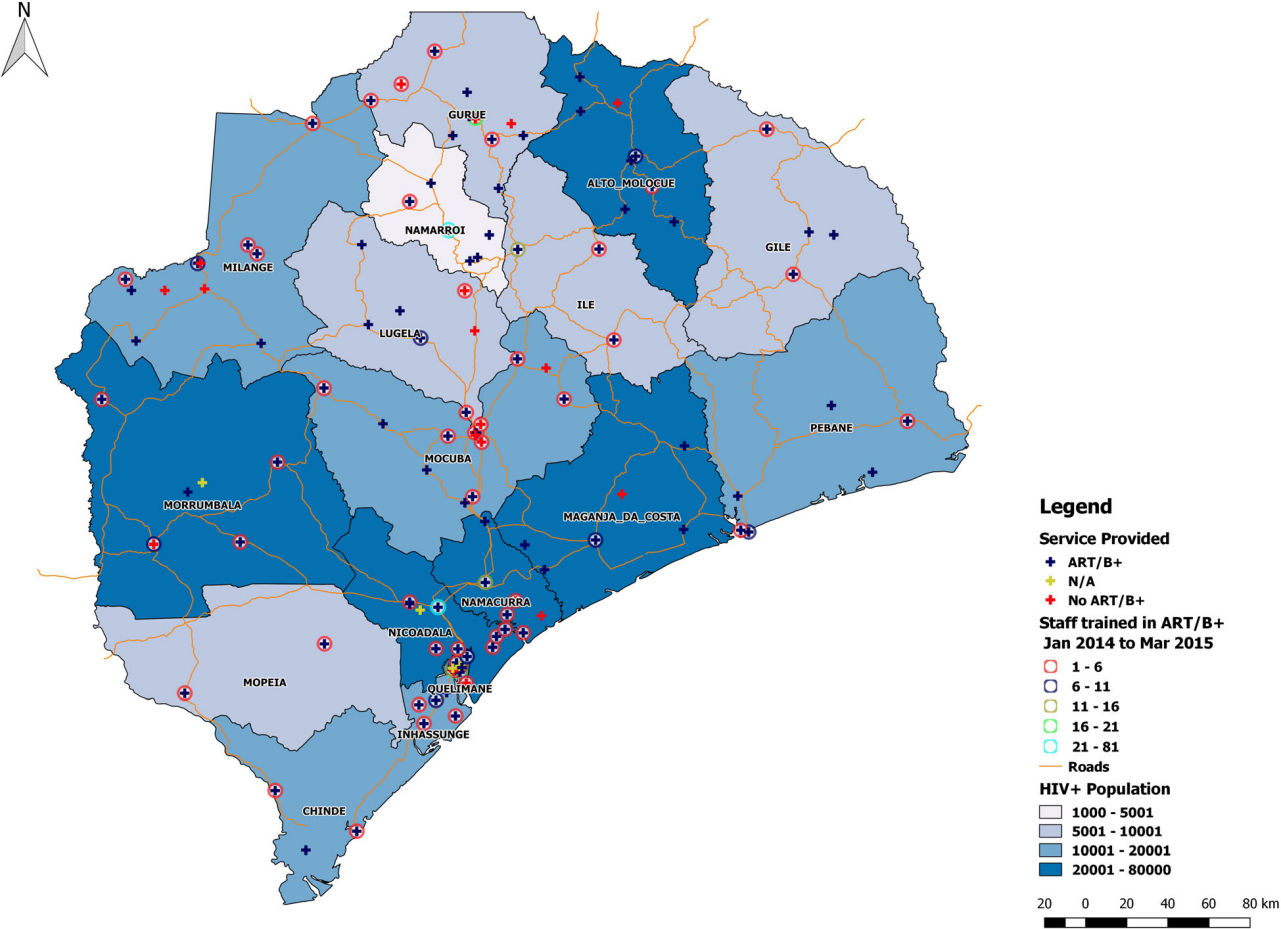
Nurses trained in ART/B+ mapped against health facilities offering ART/B+ services

The MOH and Jhpiego used data from the HRIS to examine staffing for human immunodeficiency virus/acquired immune deficiency syndrome (HIV/AIDS) services. Information on in-service training from SIFo (the HRIS in-service training database) was matched with deployment data by cadre at the facility level for Zambézia province, which is the most populous province in the country and has a high number of HIV-infected individuals [27]. The objective of the analysis was to examine the deployment of nurses trained in ART and prevention of mother-to-child transmission (PMTCT) Option B+ (lifelong ART for pregnant women) vis-à-vis the health facilities where ART and/or B+ was being provided. Figure 2 shows a map of where nurses who had received ART/B+ training were deployed (circles) against which health facilities offered ART/B+ (blue crosses). An important finding was that as of March 2015, a significant number of nurses trained in ART/B+ were deployed to facilities that did not provide any of these services—illustrated by red crosses on the circles—but that were located in districts where rapid scale-up was expected. Around 70 % of nurses trained in ART/B+ were working in ART/B+ facilities, whereas the others were deployed to facilities not presently providing these services. The information obtained through this analysis, which was subsequently conducted for other provinces such as Gaza and Inhambane, was presented to the Mozambique CDC/PEPFAR team for discussions on HRH deployment for HIV service provision.

Data from the HRIS are also being used to inform other workforce initiatives, such as allocation of new health worker graduates. A recent collaborative workforce planning project between CDC and the Atlanta-based Public Health Informatics Institute (PHII) and Georgia Institute of Technology, Jhpiego, and the MOH has led to development of a workforce allocation optimization tool that uses data from the HRIS as inputs and runs an algorithm to find the best possible match between numbers of health workers needed by cadre at each provincial location and graduates location preferences for deployment. Allocating these new health workers to both where their skills are needed and where they wish to work is expected to result in higher staff motivation and retention. The MOH piloted this optimization tool in 2015 to allocate over 1000 health graduates from many different cadres and reported the experience as positively improving the process. The development, use, and impact of the allocation tool are being documented in detail for separate forthcoming manuscripts.

#### HRIS data for HRH management

Data offered by the HRIS are starting to highlight and quantify some chronic HRH management issues that affect the health workforce and the MOH managerial and financial systems. Alerted to this in 2014, the MOH began performing more in-depth analyses on the information provided by the HRIS in order to better identify the magnitude of the issues and explore solutions. Some of the most relevant findings relate to the following categories: (i) employment status, (ii) staffing efficiencies, and (iii) retirement.

##### Employment status

The process for absorbing health workers onto government payroll, known as nomination, is lengthy. Workers must spend 2 years in probation status until they are eligible to become permanent government employees and attain definitive nomination. During the probation period, workers receive limited benefits (e.g., cannot be promoted or ask for educational leave). Becoming a permanent employee requires an MOH manager action approving the employee’s paperwork documenting completion of probation.

Analysis of the data in the HRIS revealed that almost 10 000 workers (25 % of the entire MOH health workforce) had passed the 2-year probation period but not been updated as definitive in the MOH information systems [28]. To address this issue, in August 2014, the MOH started a campaign to update the status of the employees and accurately reflect it in the HRIS. For future monitoring of employee status, the MOH established a system of alerts in semi-monthly reports:

- No alert: more than 3 months until the 2-year limit for probation
- Green: 3 months or less until the 2-year limit
- Yellow: worker has exceeded the 2-year limit for probation by up to 6 months
- Red: worker has exceeded the 2-year limit for probation for more than 6 months

Another important employment status issue that has emerged involves promotions and salary levels. After 2 years’ service as a definitive employee, all workers are due an automatic promotion which includes a salary increase. However, analysis of HRIS data identified more than 10 000 health workers that started employment over 2 years ago but were never promoted. HRIS data showed this occurred across all provinces, though two of them (Nampula and Zambézia) accounted for nearly 30 % of the total.

##### Staffing efficiencies

Another area that the HRIS has informed is unpaid leave planning (see Table 1).

**Table 1:** The three types of unpaid leave for nominated workers in the Mozambican public sector

Leave type	Conditions of the unpaid leave	No. of workers
Unlimited	Indefinite period of time, worker might not have a job to return to	599
Registered	6 months to 1 year, worker’s same position is held	354
Special	Indefinite, for special studies (master’s or doctoral level)	28

As of August 2014, the HRIS found 981 health workers presently on unpaid leave, which represents about 2.5 % of the total Mozambique health workforce [28]. Of these, 354 were on registered leave. At present, the database cannot calculate the duration individuals have been on unpaid leave, but adding the start and end dates to the electronic record will facilitate this analysis in the future and assist the MOH to determine where action is needed on an individual basis. Provinces with more workers on unlimited leave than the average were Cabo Delgado, Tete, Gaza, Maputo Province, Maputo City, and Central MOH. The latter three (the capital region) accounted for 37 % of the total.

Another staffing issue preliminarily identified by the HRIS is the difference between a health worker’s pay point (the location of the budget from which their salary is paid) and the location where the employee actually works. The HRIS has enabled the identification of 165 health workers physically working in one location whose salary was being paid with funds from another location. For instance, the salaries of 31 health workers at Maputo Central Hospital, 39 at Maputo City, and 13 working in the Central MOH were being paid with funds from other provinces. At the same time, Maputo City and Maputo Province funds were being used to pay the salaries of more than 20 health workers in other locations. Funds from Niassa province, one of the most underserved, were paying for 45 health workers who were working elsewhere.

##### Retirement

If a worker’s retirement documents have not been processed when the worker reaches mandatory retirement age, they will cease to work but continue to receive their full salary. They are deemed “desligados” (inactive). It can take from 6 months to over a year to process a worker’s retirement, as it requires evidence of their 35 years of service and depends on processing and decision making at the central government level.

As at August 2014, data from the HRIS showed that 1046 health workers were desligados—that is, receiving their full salary but no longer working at the facilities [28]. The estimated cost of this to the MOH is at least USD 1.5 million per annum. Although the situation occurs in almost all provinces, 41 % of the desligados were in the capital region: Maputo Province, Maputo City, and Central Hospital.

Additionally, there were 628 health workers recorded in the HRIS as actively working beyond the mandatory retirement age—which is 65 years for men and 60 years for women. Again, this situation occurred across all provinces, though two locations (Zambézia and Maputo Central Hospital) accounted for 40 % of these workers.

### Discussion

The case presentation describes experiences of the MOH and implementing partner in developing the HRIS and using data from it. We presented key findings from analyses of HRH deployment and management data in the HRIS. The finding that around 30 % of nurses trained in ART/B+ were deployed to facilities not presently providing ART/B+ services might be the result of training occurring ahead of the planned scale-up, but it could also be inefficient provision of in-service training. In the latter case, improvements can be made to align specialized skill sets with service provision, with a particular focus on districts with higher HIV prevalence. Information from the integrated HRIS databases permits this type of analysis on an ongoing basis so decision makers can ensure health workers are deployed more efficiently.

Analysis of employment status data in the HRIS identified more than 10 000 health workers that had not received their due promotions. This not only affects the current rank and salary of the health workers, it also affects the amount they are entitled to in their pensions after they retire.

Through monitoring the nearly 1000 employees recorded in the HRIS as on leave, the MOH can take steps to ensure that health workers either return from registered leave in time or that vacancies left by workers on unlimited leave for an unreasonable length of time (over 1 year) can be used to absorb new employees.

The mismatches the HRIS identified between pay points and physical working locations can occur because of the relocation of a health worker’s spouse (Mozambican law protects the family unit) or due to administrative decisions favoring a specific health worker. Facilities receiving staff paid with funds from other facilities usually accept the new personnel due to the great HRH needs they normally face. Administrative staff at facilities funding the salaries of relocated personnel continue to do so because they fear losing the budgetary allocation for the position.

HRIS data showed over 1000 staff no longer working at the health facilities but still receiving full salary (desligados). Processing retirement depends on another government sector, Social Security, to arrange pension payments. The MOH is now analyzing this situation to improve the retirement process and coordination with Social Security. It is unclear whether the health workers recorded as still actively working beyond retirement age are continuing to contribute to the health system or are already desligados.

The selected examples demonstrate some of the ways Mozambique is using HRIS data for decision making, to identify and further investigate HRH issues—and if necessary target geographical areas where they will have most impact.

The MOH and Jhpiego attribute the successful implementation of eSIP-Saúde to the following key factors, all important for the system’s sustainability:

i) Investing time in strong engagement with leadership and engendering active support from decision-making stakeholders. The extensive intersectoral collaboration between many diverse stakeholders demonstrates their united belief in the viability of the HRIS to produce results useful for decision making.
ii) Conducting a customized assessment of existing systems and procedures prior to system design. The careful approach chosen has helped to foster electronic exchange of information with other government and HRH information systems (e.g., SIFo and SIFIn) and ensure eSIP-Saúde is aligned with Mozambique’s national enterprise architecture frameworks. Increasing interoperability and combining data from different systems will enable generation of more powerful analyses and provide the evidence needed to drive decisions on health planning and policy.
iii) Building upon and linking existing MOH/GOM owned systems and infrastructure to the maximum extent possible. Developing eSIP-Saúde this way has facilitated broad acceptance by the MOH and the many stakeholders engaged at other ministries and agencies and yielded savings estimated at $367 000 in initial infrastructure and $187 000 per year in maintenance and operational costs. Over the 5 years of the project, this amounts to an estimated $1 371 000 (US dollars).
iv) Utilizing local technical support and building the capacity of the MOH (district, provincial, and national HRH managers) and CEDSIF.
v) Avoiding the proliferation of multiple partial or geographically focused HRH information systems. The HRIS approach in Mozambique has helped to stop partners duplicating efforts and to work together with defined roles.
vi) Continuous process of evolution and development through the dynamic involvement of key stakeholders. Input from the online CoP where users send alerts for required modifications enables constant validation and error correction of the system data. This provides the MOH with ownership of an evidence base that can be used to demonstrate the impact of not addressing HR issues to decision makers in other ministries. The process of retirement, for example, involves salaries, pensions, and other factors beyond MOH control and requires coordination with MEF for implementation.

The few other recent papers on HRIS in Africa describe some similar challenges, success factors, and benefits critical for improving the countries’ health systems. However, whereas both Kenya and Tanzania developed new systems [11, 17], and Uganda adopted (and adapted) the open source iHRIS software [15], Mozambique is the only documented example of a country integrating and extending existing national government systems for the HRIS. This novel approach is demonstrating rapid progress in a relatively short time and offers what may in the right setting be a faster and more cost effective alternative. This resonates with Driessen et al.’s finding that although the full cost of HRIS implementation can be daunting the benefits of HRIS investments are similar staggering in magnitude and have the advantage of being long-lasting [16]. The figures calculated for Mozambique do not take into account recurrent costs of any ghost workers and/or non-motivated workers, aspects which are to be examined in more detail in the future. As eSIP-Saúde is still being developed, further assessments are recommended, as the system matures, to provide evidence that the HRIS is stable, efficient, transparent, and sustainable in the long term.

We recommend Ministries of Health and other government sectors explore how to develop synergies among their information systems. For example, countries developing automated payroll systems could explore whether it might be used as the basis, or as a support element, for sectorial HR systems such as health. In Mozambique, lessons learned from the successful extension of the national registry of government employees (eCAF) to cover additional data elements and non-government employees for health can be used to create similar approaches for other sectors, such as education and agriculture, and build a web-based national government HR management information system.

## Conclusions

The Mozambican system is an important example of an HRIS built on a local platform with local staff. Notable models of strategic data use demonstrate that the system is empowering the MOH to improve health services delivery, health workforce allocation, and management. Combined with committed country leadership and ownership of the program, this suggests strong chances of sustainability and real impact on public health equity and quality.

## Abbreviations

APE: Community health assistant (Agente polivalentes elementares de saúde)
ART: Antiretroviral therapy
BTC: Belgian Technical Cooperation
CDC: US Centers for Disease Control and Prevention
CEDSIF: Center for Development of Financial Information Systems (Centro de Desenvolvimento de Sistemas de Informação e Finanças)
eCAF: Registry of GOM Employees (eletrônico Cadastro de Agentes e Funcionários do Estado)
eSIP-Saúde: concept name for Mozambique’s electronic integrated HRIS for health
GOM: Government of Mozambique
HCW: Health care worker
HIV/AIDS: Human immunodeficiency virus/acquired immune deficiency syndrome
HMIS: Health Management Information System
HRH: Human resources for health
HRIS: Human Resources Information System(s)
IT: Information technology
MEF: Ministry of Economy and Finance
MFL: Master Facility List
MOH: Ministry of Health (MISAU)
NGO: Non-governmental organization
PEPFAR: US President’s Emergency Plan for AIDS Relief
PMTCT: Prevention of mother-to-child transmission of HIV
SIFIn: Pre-Service Training Information System (Sistema de Informação da Formação Inicial)
SIFo: In-Service Training Information System (Sistema de Informação da Formação Continua)
SIP: Personnel Information System (Sistema de Informação de Pessoal)
TB: Tuberculosis
USG: United States Government
WHO: World Health Organization

## Footnotes

1 Figures used in the article are consistent as at time of writing. At time of publication, the number of public sector HCWs (paid from the state budget and Pro Saude donor funds) has increased to 52 213.

2 Proof of life is verification, in accordance with Mozambican law, that a government health worker is alive. The law mandates all government employees to periodically present themselves before a government official at designated locations.
